# Larvicidal activity and possible mode of action of four flavonoids and two fatty acids identified in *Millettia pinnata* seed toward three mosquito species

**DOI:** 10.1186/s13071-015-0848-8

**Published:** 2015-04-19

**Authors:** Haribalan Perumalsamy, Myung Jin Jang, Jun-Ran Kim, Murugan Kadarkarai, Young-Joon Ahn

**Affiliations:** Research Institute of Agriculture and Life Sciences, Seoul National University, Seoul, 151-921 Republic of Korea; Department of Agricultural Biotechnology, Seoul National University, Seoul, 151-921 Republic of Korea; National Academy of Agricultural Science, Rural Development Administration, Wanju, 565-851 Jeollabuk-do Republic of Korea; Department of Zoology, Bharathiar University, Coimbatore, 641046 Tamil Nadu India

**Keywords:** *Millettia pinnata*, Fabaceae, Seed, Natural mosquito larvicide, Flavonoids, Fatty acids, Acetylcholinesterase inhibition, Octopaminergic receptor

## Abstract

**Background:**

*Aedes aegypti* and *Aedes albopictus* and *Culex pipiens pallens* mosquitoes transmit dengue fever and West Nile virus diseases, respectively. This study was conducted to determine the toxicity and mechanism of action of four flavonoids and two fatty acids from *Millettia pinnata* (Fabaceae) seed as well as six pure fatty acids and four fatty acid esters toward third instar larvae from insecticide-susceptible *C. pipiens pallens* and *A. aegypti* as well as wild *A. albopictus*. Efficacy of 12 experimental liquid formulations containing *M. pinnata* seed methanol extract and hydrodistillate (0.5–10.0% liquids) was also assessed.

**Methods:**

The contact toxicities of all compounds and 12 formulations were compared with those of two larvicides, temephos and fenthion and the commercial temephos 200 g/L emulsifiable concentrate (EC). The possible mode of larvicidal action of the constituents was elucidated using biochemical methods. Larval mortality and cAMP level were analyzed by the Bonferroni multiple-comparison method.

**Results:**

Potent toxicity was produced by karanjin, oleic acid, karanjachromene, linoleic acid, linolenic acid, pongamol, pongarotene, and elaidic acid toward *C. pipiens pallens* larvae (24 h LC_50_, 14.61–28.22 mg/L) and *A. aegypti* larvae (16.13–37.61 mg/L). Against wild *A. albopictus* larvae, oleic acid (LC_50_, 18.79 mg/L) and karanjin (35.26 mg/L) exhibited potent toxicity. All constituents were less toxic than either temephos or fenthion. Structure–activity relationship indicates that the degree of saturation, the side chain length, and the geometric isomerism of fatty acids appear to play a role in determining the fatty acid toxicity. Acetylcholinesterase (AChE) is the main site of action of the flavonoids, oleic acid, and palmitic acid. The mechanism of larvicidal action of elaidic acid, arachidic acid, and behenic acid might be due to interference with the octopaminergic system. Linoleic acid and linolenic acid might act on both AChE and octopaminergic receptor. *M. pinnata* seed extract or hydrodistillate applied as 10% liquid provided 100% mortality toward the three mosquito species larvae and the efficacy of the liquids was comparable to that of temephos 200 g/L EC.

**Conclusion:**

Further studies will warrant possible applications of *M. pinnata* seed-derived products as potential larvicides for the control of mosquito populations.

**Electronic supplementary material:**

The online version of this article (doi:10.1186/s13071-015-0848-8) contains supplementary material, which is available to authorized users.

## Background

The yellow fever mosquito, *Aedes aegypti* (Linnaeus 1762) [1], the Asian tiger mosquito, *Aedes albopictus* (Skuse 1894) [2], and the northern house mosquito, *Culex pipiens pallens* (Coquillett 1898) [3], are serious disease vectoring insect pests because of their widespread distribution and abundance worldwide [4]. More than 2.5 billion people are at risk of dengue infection over 100 countries worldwide, and there may be 50–100 million dengue infections every year, including 22000 deaths annually, mostly among children [5]. A recent study calculated that 3.97 billion people are at risk of dengue infection in 128 countries worldwide [6,7]. From 1999 to 2010, 37088 cases of human West Nile virus disease (including 16196 neuroinvasive disease cases) were reported in the United States (US), resulting in 1549 deaths [8]. With global warming, increased international travel, and tainted fresh water pools, a number of mosquitoes are distinctly increasing in incidence with a high occurrence of dengue fever all over the globe [9,10]. Widespread insecticide resistance [11] has been a major obstacle in the cost-effective integrated mosquito management program. In addition, the number of approved insecticides may be reduced in the near future in the US [12] and in the European Union [13] because of re-registration of conventional insecticides. The removal of conventional insecticide products from markets due to the increase in insecticide resistance or other concerns will have a serious impact on the proliferation of mosquitoes. There is a pressing need for the development of selective alternatives for the control of mosquitoes, with novel target sites to establish a rational management strategy and tactics because vaccines for malaria [14] or dengue [15] are not yet available.

Plants have been suggested as alternative sources for conventional mosquito larvicides largely because they constitute a potential source of bioactive secondary substances that have been perceived by the general public as relatively safe and with less risk to the environment, and with minimal impacts to animal and human health [16-18]. Secondary substances often act at multiple and novel target sites [18-20], thereby reducing the potential for resistance [21,22]. They are regarded as potential sources for developing commercial insecticides as certain plant preparations and their constituents meet the criteria as minimum-risk insecticides [23]. Previous studies have shown that a methanol extract from the seeds of Indian beech, *Millettia pinnata* (L.) Panigrahi (Fabaceae) (formerly *Pongamia pinnata* (L.) Pierre), possessed good larvicidal activity toward *C. pipiens pallens* and *A. aegypti*. No information is available concerning the potential use of *M. pinnata* seed-derived materials for managing mosquitoes for future commercialization, although phytochemistry, pharmacological activities, and traditional and folk medicine uses of the plant have been well documented by Arote and Yeole [24] and Meera et al. [25].

The aim of the study was to assess the contact toxicity of the four flavonoids (karanjin, karanjachromene, pongamol, and pongarotene) and two fatty acids (oleic acid and palmitic acid) from *M. pinnata* seed extract and the five organic pure fatty acids from the seed hydrodistillate and one structurally related compound (elaidic acid), as well as two fatty acid methyl esters and two fatty acid ethyl esters to third instar larvae from insecticide-susceptible *C. pipiens pallens* and *A. aegypti*, as well as a wild colony of *A. albopictus*. Results were compared with those of the two conventional larvicides temephos and fenthion. The efficacy of 12 liquid formulations (0.5–10%) containing the seed extract or hydrodistillate were compared with those of the commercial larvicide temephos 200 g/L emulsifiable concentrate (EC) because the larvicide is known to have low toxicity to mammals and aquatic organisms and is less persistent in the environment [26]. Quantitative structure–activity relationship (QSAR) of the test compounds is also discussed. In addition, the possible mode of larvicidal action of the constituents was elucidated using biochemical methods.

## Methods

### Instrumental analysis

^1^H and ^13^C NMR spectra were recorded in CDCl_3_ on an AVANCE 600 spectrometer (Bruker, Rheinstetten, Germany) at 400 and 100 MHz, respectively, using tetramethylsilane as an internal standard, and chemical shifts are given in δ (ppm). Distortionless enhancement by polarization transfer spectra was acquired using the Bruker software. UV spectra were obtained in methanol or acetone on a UVICON 933/934 spectrophotometer (Kontron, Milan, Italy), and mass spectra on a JMS-DX 303 spectrometer (Jeol, Tokyo, Japan). Silica gel 60 (0.063–0.2 mm) (Merck, Darmstadt, Germany) was used for column chromatography. Merck precoated silica gel plates (Kieselgel 60 F_254_) were used for analytical thin-layer chromatography (TLC). An Agilent 1200 series high-performance liquid chromatography (HPLC) (Agilent, Santa Clara, CA) was used for isolation of active principles.

### Materials

Eight fatty acids and four fatty acid esters used in this study were as follows: palmitic acid (C16:0), stearic acid (C18:0), oleic acid (C18:1n9), linoleic acid (C18:2n6), linolenic acid (C18:3n3), methyl oleate, ethyl oleate, methyl linoleate, and ethyl linoleate purchased from Sigma-Aldrich (St. Louis, MO); arachidic acid (C20:0), behenic acid (C22:0), and elaidic acid (C18:1n9) purchased from Tokyo Chemical Industry (Tokyo). The organophosphorus (OP) insecticides temephos (97.3% purity) and fenthion (98.4% purity) were purchased from Riedel (Seelze, Germany) and Supelco (West Chester, PA), respectively. Commercial temephos 200 g/L EC was supplied by Pharmcle (Seoul, Republic of Korea (ROK)). Ethoxylated castor oil + polyoxyethylene dodecyl mono ether, a surfactant, was a gift from Hannong Chemical (Anyang, Gyeonggi, ROK). Acetylthiocholine iodide (ATChI), 5, 5'-dithio-bis(2-nitrobenzoate) (DTNB), eserine salicylate, and octopamine were purchased from Sigma-Aldrich. Bovine serum albumin (BSA) and cyclic AMP (cAMP) Biotrak Enzyme immunoassay system were purchased from Sigma-Aldrich and GE Healthcare (Little Chalfont, Buckinghamshire, UK), respectively. Mouse monoclonal antibody and horseradish peroxidase-labeled cAMP were purchased from R&D Systems (Minneapolis, MN). All of the other chemicals and reagents used in this study were of analytical grade quality and available commercially.

### Mosquitoes

The stock cultures of *C. pipiens pallens* and *A. aegypti* [27] have been maintained in the laboratory without exposure to any known insecticide. Engorged *A. albopictus* females were collected near rice paddy fields and cowsheds in Daejeon (ROK) in early August, 2011 using black light FL-6w traps (Shinyoung, Seoul). The rice paddy fields and cow sheds had varying histories of insecticide use. They have been separately maintained in temperature-controlled insect rearing rooms (Seoul National University) to prevent cross-contamination. Larvae were reared in plastic trays (24 × 35 × 5 cm) containing 0.5 g of sterilized diet (40-mesh chick chow powder/yeast, 1/1 by weight). Adults were maintained on a 10% sucrose solution and blood fed on live mice. All stages were held at 27 ± 1°C and 65–75% relative humidity under a 16:8 h light:dark cycle. The wild mosquitoes were reared for four generations to ensure sufficient numbers for testing.

### Plant material

Seeds of *M. pinnata* were collected from the foothill at Western Ghats (11°22′30′′N, 76°45′30′′E) (Coimbatore, Tamil Nadu, India) in September 2009. A certified botanical taxonomist was used to identify the plant. A voucher specimen (MP–01) was deposited in the Research Institute of Agriculture and Life Sciences, Seoul National University.

### Hydrodistillation

Seeds (500 g) of *M. pinnata* were pulverized and subjected to hydrodistillation at 100°C for 2 h using a Clevenger-type apparatus. The volatile oil was dried over anhydrous sodium sulfate and stored in a sealed vial at 4°C until use. The yield of the hydrodistillate from the seeds was 1.87%. The seed hydrodistillate was used for fatty acid analysis and experimental formulations.

### Experimental liquid formulations

Twelve experimental liquid formulations containing *M. pinnata* seed methanol extract (MPS-ME) and seed hydrodistillate (MPS-HD) were prepared to determine the effective larvicide products. The 0.5, 1, 2.5, 5, 7.5, and 10% liquid formulations were, respectively, composed of 0.5, 1, 2.5, 5, 7.5, and 10% of the corresponding extract or hydrodistillate, 2% surfactant (ethoxylated castor oil + polyoxyethylene dodecyl mono ether), 5% ethanol, and sterile distilled water at 92.5, 92, 90.5, 88, 85.5, and 83%.

### Extraction and isolation

Air-dried seeds (260 g) of *M. pinnata* were pulverized, extracted with methanol (2 × 6 L) at room temperature for 1 day, and filtered. The combined filtrate was concentrated to dryness by rotary evaporation at 40°C to yield ~46.42 g of a pale yellowish tar. The extract (40 g) was sequentially partitioned into hexane- (6.44 g), chloroform- (5.92 g), ethyl acetate- (1.0 g), butanol- (2.06 g), and water-soluble (24.58 g) portions for subsequent bioassay. The organic solvent-soluble portions were concentrated under vacuum at 40°C, and the water-soluble portion was freeze-dried. For isolation of active principles, 50 mg/L of each *M. pinnata* seed-derived material was tested in a direct-contact mortality bioassay, as described previously [28].

**Figure 1:**
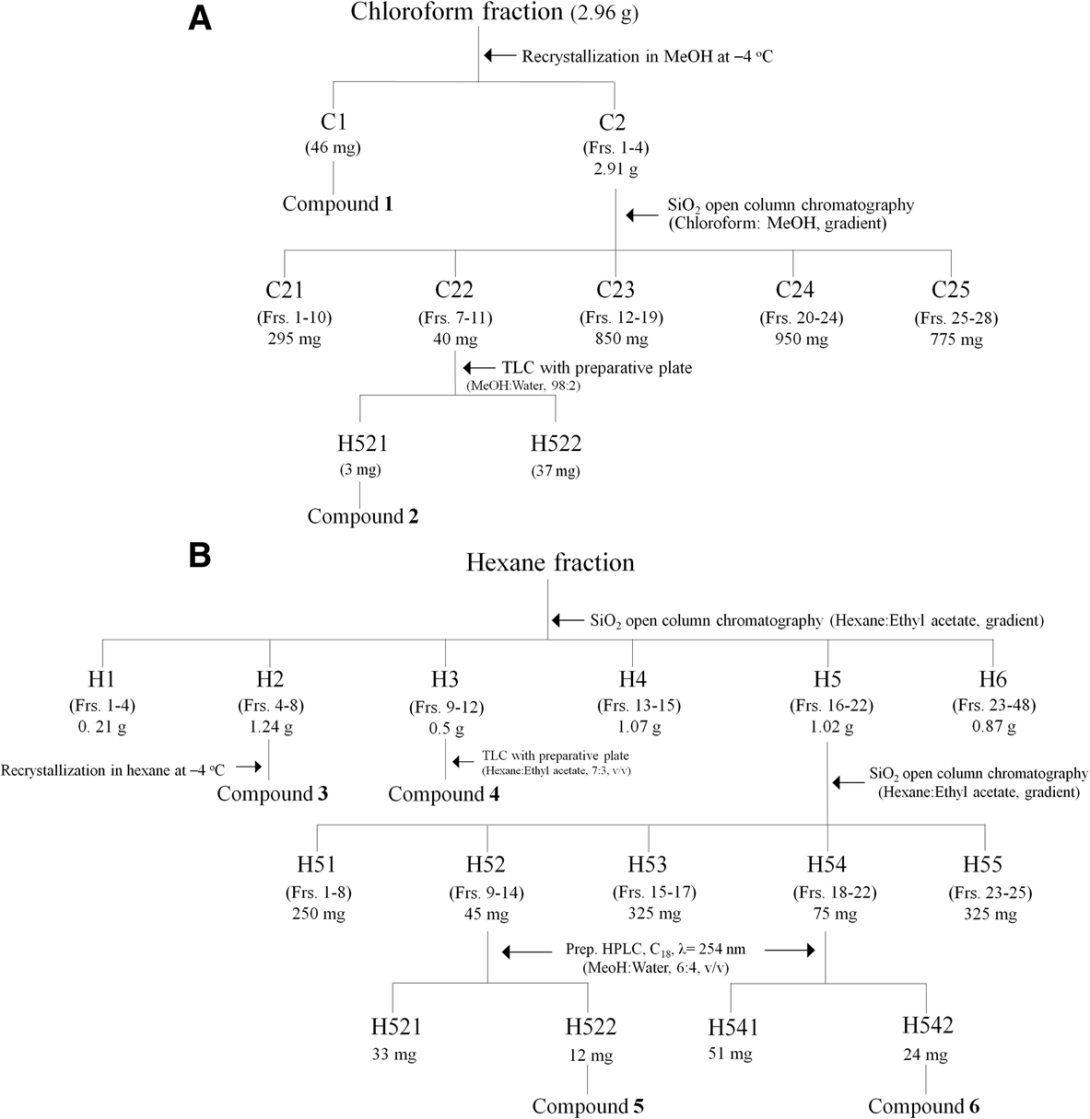
Isolation procedures of larvicidal principles. The *Millettia pinnata* seed methanol extract was sequentially partitioned into hexane-, chloroform-, ethyl acetate-, butanol-, and water-soluble portions. For isolation of active principles from the chloroform-soluble fraction **(A)** and hexane-soluble fraction **(B)**, 50 mg/L of each *M. pinnata* seed-derived material was tested in a direct-contact mortality bioassay toward third instar larvae from *Culex pipiens pallens* and *Aedes aegypti*.

The chloroform-soluble fraction (2.96 g) was most biologically active (Table 1) and was recrystallized in methanol at −4°C to afford compound 1 (46 mg) (Figure 1A). The remaining portion (2.91 g) was chromatographed on a 70 × 5.5 cm silica gel (300 g) column by elution with a gradient of chloroform and methanol [(100:0 (1 L), 99:1 (2 L), 98:2 (2 L), 95:5 (2 L), 90:10 (2 L), 70:30 (1 L), and 0:100 (1 L) by volume] to provide 28 fractions (each about 250 mL). Column fractions were monitored by TLC on silica gel plates developed with chloroform and methanol (98:2 by volume) mobile phase. Column fractions with similar *R*_f_ values on the TLC plates were pooled. Spots were detected by spraying with 2% H_2_SO_4_ and then heating on a hot plate. Active fractions 7 to 11 (40 mg) were pooled and purified by preparative TLC plates developed with chloroform and methanol (98:2 by volume) to provide compound **2** (3 mg, *R*_f_ = 0.61). The other active hexane-soluble fraction (5 g) was chromatographed on a 70 × 5.5 cm silica gel (300 g) column by elution with a gradient of hexane and ethyl acetate [(10:1 (2.2 L), 9:1 (2 L), 7:3 (2 L), 5:5 (1 L), and 3:7 (1 L) by volume] and finally with methanol (1 L) to provide 48 fractions (each about 250 mL) (Figure 1B). Column fractions were monitored by TLC on silica gel plates developed with hexane and ethyl acetate (7:3 by volume) mobile phase. Fractions with similar *R*_f_ values on the TLC plates were pooled, as stated previously. Three active fractions 4 to 8 (H2, 1.24 g), 9 to 12 (H3, 590 mg), and 16 to 22 (H5, 1.02 g) were obtained. Fraction H2 was recrystallized in hexane at −4°C to afford compound 3 (78 mg). Fraction H3 was purified by preparative TLC plates developed with hexane and ethyl acetate (7:3 by volume) to yield compound 4 (85 mg, *R*_f_ = 0.61). Fraction H5 was rechromatographed on a 70 × 5.5 cm silica gel (300 g) column by elution with a gradient of hexane and ethyl acetate [(10:1 (2.2 L), 9:1 (2 L), 7:3 (2 L), 5:5 (1 L), and 3:7 (1 L) by volume] and finally with methanol (1 L) to provide 25 fractions (each about 150 mL). A preparative HPLC was used for separation of the constituents from the active fractions 9 to 14 (H52, 45 mg) and 18 to 22 (H54, 75 mg). The column was a 7.8 mm i.d. × 300 mm Waters μBondapak C18 (Milford, MA) with a mobile phase of methanol and water (6:4 by volume) at a flow rate of 1 mL/min. Chromatographic separations were monitored using a UV detector at 254 nm. Finally, two active principles **5** (12 mg) from fraction H52 and **6** (24 mg) from fraction H54 were isolated at a retention time of 19.30 and 31.22 min, respectively.

**Table 1:** **Toxicity of each fraction obtained from the solvent partitionings of the methanol extract of** ***Millettia pinnata*** **seeds and seed hydrodistillate to third instar larvae from insecticide-susceptible** ***Culex pipiens pallens*** **and** ***Aedes aegypti*** **and wild** ***Aedes albopictus*** **during a 24 h exposure**

**Material**	**LC**_**50**_ **, mg/L (95% CL)**		
	***C. pipiens pallens***	***A. aegypti***	***A. albopictus***
Methanol extract	24.19 (20.67–28.59)	27.70 (24.53–31.82)	61.30 (54.75–69.79)
Hexane-soluble fr.	19.81(17.35–22.66)	22.21(19.34–25.08)	54.70 (50.57–60.10)
Chloroform-soluble fr.	12.40 (10.39–14.96)	14.51 (12.41–16.77)	43.83 (40.01–48.40)
Ethyl acetate-soluble fr.	73.27 (69.24–78.23)	75.29 (71.27–80.50)	77.21 (73.36–82.28)
Butanol-soluble fr.	95.95 (92.07–100.91)	97.60 (93.54–103.04)	134.58 (126.14–146.18)
Water-soluble fr.	>200	>200	>200
Hydrodistillate	27.38 (22.83–32.46)	32.57 (27.20–38.80)	47.99 (39.10–60.79)

### Fatty acid analysis of *Millettia pinnata* seed hydrodistillate

Because MPS-HD contains various free fatty acids with low volatility [29,30], fatty acid methyl esters (FAMEs) were prepared following the method described previously by Rafael and Mancha [31]. In brief, 1 μg of MPS-HD was methylated overnight at 25°C with diazomethane in 200 μL of diethyl ether. Solvent and excess diazomethane were removed under a stream of N_2_, and the residue was redissolved in diethyl ether for gas chromatography (GC) analysis.

GC of the FAMEs was performed using an Agilent 7890A gas chromatograph (Agilent, Palo Alto, CA) equipped with a split injector and a flame ionization detection (FID) system. Constituents were separated with a 30 m × 0.25 mm i.d. (*d*_f_ = 0.25 μm) DB-wax capillary column (Agilent J&W Scientific, Folsom, CA). The oven temperature was kept at 50°C (1 min isothermal) and programmed to 200°C at a rate of 25°C/min, and then 230°C at a rate of 3°C/min (held for 6 min at final temperature). The linear velocity of the nitrogen carrier gas was 36 cm/s (at 50°C) at a split ratio of 50:1. The constituents were identified by coelution of authenticated samples following coinjection, and their composition was calculated to percentage content based on peak area.

### Gas chromatography–mass spectrometry of methylated samples

The gas chromatography–mass spectrometry (GC-MS) analysis of methylated samples was performed using a HP 6890 gas chromatograph-JMS-600 W mass spectrometer for further identification of fatty acids on the GC-FID chromatogram. The capillary column and temperature conditions for the GC-MS analysis were the same as those stated previously for GC analysis. Helium carrier gas was used at a column head pressure of 15.7 psi (39.2 kPa). The ion source temperature was 230°C, and mass spectra were obtained in EI-scan mode at 70 eV electron energy.

### Bioassay

A direct-contact mortality bioassay [28] was used to evaluate the toxicity of all compounds to third instar larvae from the susceptible and wild mosquito populations. Each compound in acetone (for karanjin) or methanol (for the other compounds) was suspended in distilled water with Triton X-100 (20 μL/L). Groups of 20 mosquito larvae were separately put into paper cups (270 mL) containing each test compound solution (250 mL). The OPs temephos and fenthion served as positive controls and were similarly formulated. Negative controls consisted of the acetone– or methanol–Triton X-100 solution in distilled water. Based on the preliminary test results, the toxicity of each compound and insecticide was determined with four to six concentrations ranging from 0.1 to 200 mg/L and 0.001 to 0.1 mg/L, respectively. Treated and control (acetone– or methanol–Triton X-100 solution only) larvae were held under the same conditions as those used for colony maintenance. At 24 h post-treatment, a larva was considered dead if it did not move when prodded with a fine wooden dowel [28]. All treatments were replicated three times using 20 larvae per replicate.

In separate experiments, the efficacy of the 12 experimental liquid formulations was evaluated, as stated previously. Groups of 20 mosquito larvae were separately put into paper cups containing each test solution. Temephos 200 g/L EC served as a positive control. Negative controls consisted of the ethoxylated castor oil + polyoxyethylene dodecyl mono ether solution in distilled water or water. Mortalities were recorded, as stated previously. All treatments were replicated three times using 20 larvae per replicate.

### Acetylcholinesterase assay

Third instar larvae of *A. aegypti* was used in all experiments. Whole bodies of the larvae (14.5 mg fresh weight/1 mL buffer) were homogenized in 2 mL of ice-cold 0.1 M phosphate buffer (pH 8.0) using a Teflon glass tissue homogenizer. After filtering through cheese cloth, the homogenate was centrifuged at 1000 *g* at 4°C for 5 min. The supernatant was used as the acetylcholinesterase (AChE) preparation. Protein content was determined by the Bradford dye method [32] using BSA as the standard. Microplate AChE assay was carried out following the method of Hemingway et al. [33] adapted from Ellman et al. [34]. The reaction mixture consisted of 50 μL of the crude enzyme preparation, 150 μL of 0.1 M phosphate buffer, 20 μL of 3 mM DTNB in phosphate buffer (pH 7.0), and 1 μL of various concentrations of each test compound in ethanol. The reaction mixture was incubated at 30°C for 5 min and 20 μL of 32 mM ATChI was then added to the mixture. After incubation for 30 min, the reaction was stopped by adding 20 μL of 5 mM eserine salicylate. The absorbance was recorded at 412 nm using a VersaMax microplate reader (Molecular Devices, Sunnyvale, CA). Results were expressed as mean ± SE of triplicate samples of three independent experiments.

### Determination of cyclic AMP level

The *in vitro* octopamine sensitive adenylate cyclase activity was investigated according to the modified method of Pratt and Pryor [35]. The whole bodies of 75 third instar *A. aegypti* larvae (106.2 mg) were homogenized in 500 μL of 2 mM Tris-maleate buffer (pH 7.4) containing 0.8 mM ethylene glycol tetraacetic acid (EGTA). Adenylate cyclase activity was measured using a cAMP Biotrak Enzyme immunoassay system according to the manufacturer’s instructions. The assay was conducted in a total volume of 100 μL containing 80 mM Tris-maleate buffer, 5 mM theophylline (to inhibit phosphodiesterase activity), 2 mM MgSO_4_, 0.5 mM adenosine triphosphate (ATP), 0.2 mM EGTA, 50 μL of whole body homogenate (equivalent to 4.22 μg protein), and 1 μL of the test compounds in Tris-maleate buffer containing 0.2% ethanol. After incubation for 5 min at 20°C, the reaction was initiated by the addition of ATP. Incubation was carried out at 30°C for 3 min in a shaking water bath. The reaction was terminated by boiling for 2 min, and then the assay tube was cooled and centrifuged for 10 min at 8000 *g*. The 50 μL aliquots of the supernatant were assayed for cAMP level.

The polystyrene microplates (1 strip of 8 wells) coated with a goat anti-mouse polyclonal antibody were used. Fifty microliters of primary antibody (mouse monoclonal antibody) solution was added to each well except the blank wells (or the nonspecific binding (NSB) wells). The wells covered with the adhesive strip were incubated for 1 h at 25°C in a shaking incubator (480 rpm), followed by four times washing steps, each with 400 μL of wash buffer. The 50 μL of the test samples for cAMP determination and cAMP standard were added to wells. Control, blank (NSB), and zero standard wells were added with 50 μL of the diluent RD5-55 buffer. The 50 μL of cAMP conjugate (horseradish peroxidase-labeled cAMP) was then added to wells. The plate covered with a new adhesive strip was incubated for 2 h at 25°C on the shaker, followed by the four times washing steps. Then, 200 μL of substrate solution (equal volume of stabilized hydrogen peroxide and stabilized chromogen) was added to each well, and the test plate was incubated for 30 min at 25°C on the benchtop in darkness. Finally, the reaction was stopped by adding 100 μL of stop solution (2 N sulfuric acid) to each well. Optical densities at 450 and 540 nm were measured using the VersaMax microplate reader. The readings at 540 nm were subtracted from the readings at 450 nm. The cAMP concentrations were expressed as nmol/μg protein. Results were expressed as mean ± SE of duplicate samples of three independent experiments.

### Data analysis

Data were corrected for control mortality using Abbott’s formula [36]. The percentages of mortality were transformed to arcsine square-root values for analysis of variance (ANOVA). The concentration of the test compounds required to produce 50% inhibition of AChE activity (IC_50_) was determined using a SAS 9.13 program [37]. IC_50_ values and cAMP levels were subjected to ANOVA. The Bonferroni multiple-comparison method was used for comparison of means [37]. Means ± SE of untransformed data are reported. Concentration–mortality data were subjected to probit analysis [37]. The LC_50_ values for each species and their treatments were considered to be significantly different from one another when their 95% confidence limits (CLs) did not overlap.

Ethical approval was obtained from the Institutional Animal Care and Use Committees of Seoul National University for this study.

## Results

### Chemical composition of *Millettia pinnata* seed hydrodistillate

Methylation of fatty acids in MPS-HD showed that the seed hydrodistillate consists of five major fatty acids (>3.0%) and four minor fatty acids by comparison of mass spectral data and coelution of authenticated samples following coinjection (Table 2). The five major constituents were oleic acid, linoleic acid, palmitic acid, stearic acid, and behenic acid, and comprised 38.7, 11.0, 8.5, 6.6, and 3.4% of the seed hydrodistillate, respectively.

**Table 2:** **Chemical composition of** ***Millettia pinnata*** **seed hydrodistillate**

**Compound**	**Lipid Number**	**RT (min)**	**% area in GC-FID**	**Identification**	
				**CI**	**MS**
Palmitic acid^*^	C16:0	10.20	8.5	O	O
Stearic acid^*^	C18:0	11.35	6.6	O	O
Oleic acid^*^	C18:1n9	11.52	38.7	O	O
Linoleic acid^*^	C18:2n6	11.85	11.0	O	O
Linolenic acid^*^	C18:3n3	12.37	1.8	O	O
Arachidic acid^*^	C20:0	12.97	1.3	O	O
Gadoleic acid	C20:1	13.20	0.8	Χ	O
Behenic acid^*^	C22:0	15.53	3.4	O	O
Lignoceric acid	C24:0	19.74	0.8	Χ	O

RT, retention time; GC-FID, gas–liquid chromatography with flame ionization detection; CI, coinjection with authentic sample in GC-FID analysis; MS, mass spectrometry.

^*^Major constituent (>3%).

### Bioassay-guided fractionation and identification

**Figure 2:**
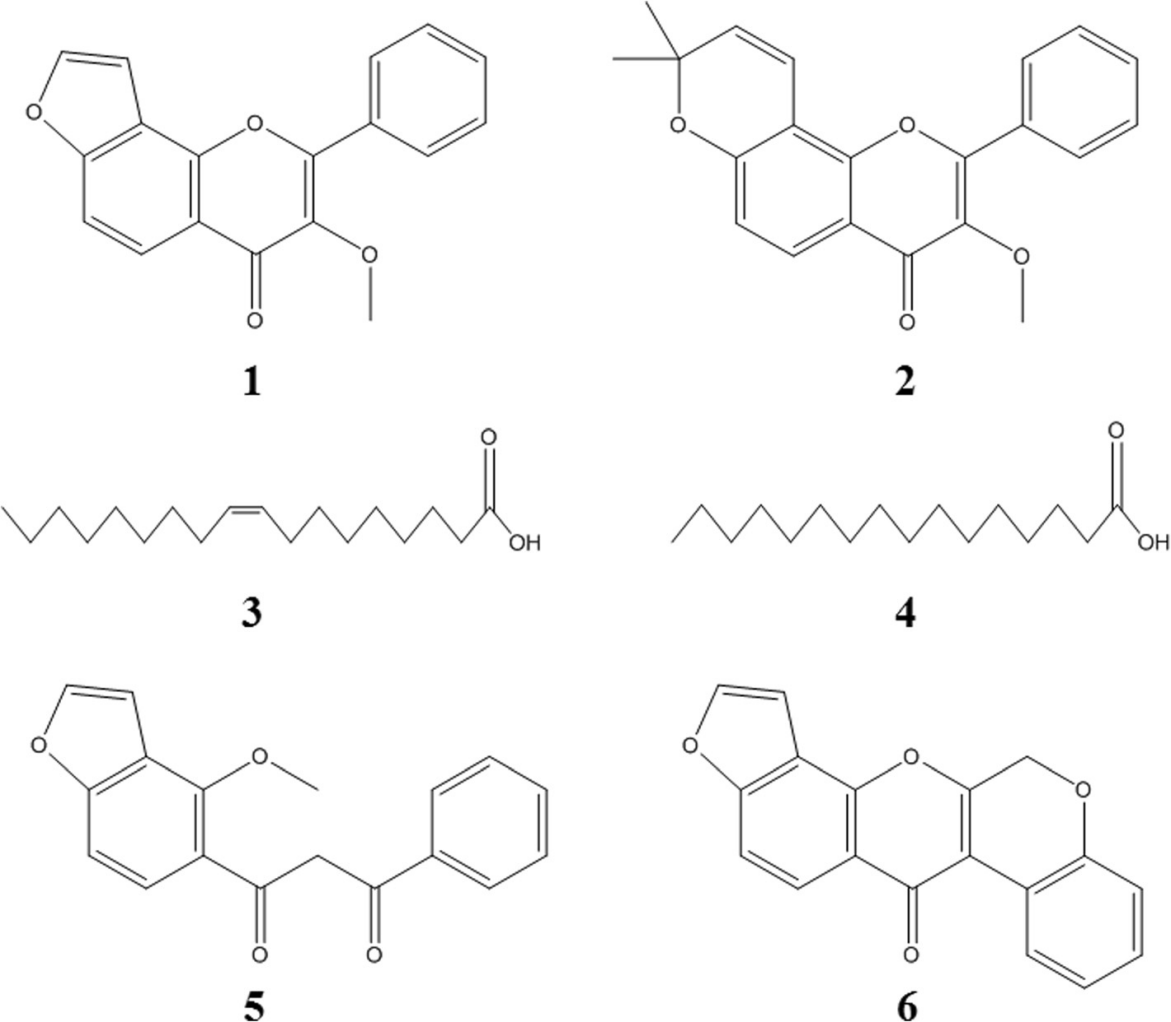
Structures of isolated compounds. The furanoflavonoid karanjin (**1**), the pyranoflavonoid karanjachromene (**2**), the unsaturated fatty acid oleic acid (**3**), the saturated fatty palmitic acid (**4**), the dihydrochalcone flavonoid pongamol (**5**), and the rotenoid flavonoid pongarotene (**6**). These constituents are identified in *Millettia pinnata* seed in this study. The chemical formulae of these compounds are C_18_H_12_O_4_ (**1**), C_21_H_18_O_4_ (**2**), C_18_H_34_O_2_ (**3**), C_18_H_32_O_2_ (**4**), C_18_H_14_O_4_ (**5**), and C_18_H_10_O_4_ (**6**); the molar masses are 292.29, 334.36, 282.46, 256.42, 294.32, and 291.06 g/mol.

Direct-contact mortality bioassay-guided fractionation of *M. pinnata* seed extract afforded six active principles identified by spectroscopic analyses, including MS and NMR. The six larvicidal principles were characterized as karanjin (1), karanjachromene (2), oleic acid (3), palmitic acid (4), pongamol (5), and pongarotene (6) (Figure 2) by spectroscopic analyses, including MS and NMR. Karanjin (1) was identified on the basis of the following evidence: white crystal. UV (acetone): λ_max_ nm = 215, 340. EI-MS (70 eV), *m*/*z* (% rel. int.): 292 [M]^+^ (100), 273 (12), 263 (7), 160 (66), 149 (7), 125 (12), 97 (19), 61 (49). ^1^H NMR (CDCl_3_, 600 MHz): δ 3.94 (3H, s), 6.85, (1H, d *J* = 1.4 Hz) 7.43 (1H, d, *J* = 1.4 Hz), 7.59 (3H, m), 7.67 (1H, d, *J* = 8.8 Hz), 8.08 (1H, d, *J* = 2.1 Hz), 8.11 (1H, d, *J* = 8.8 Hz), 8.21 (1H, m) (Additional file 1). ^13^C NMR (CDCl_3_, 150 MHz): δ 60.3 q, 105.3 d, 110.7 d, 118.2 s, 120.7 s, 122.4 d, 127.9 d, 128.0 d, 129.3 d, 129.5 d, 130.5 d, 131.5 s, 132.2 s, 142.6 d, 151.0 s, 155.4 s, 159.1 s, 174.9 s (Additional file 2). Karanjachromene (**2**): white crystal. UV (EtOH): λ_max_ nm = 260, 320. EI-MS (70 eV), *m*/*z* (% rel. int.): 334 [M]^+^ (64), 319 (100), 291 (21), 263 (19), 175 (33), 160 (13), 127 (5), 97 (17), 71 (21). ^1^H NMR (CDCl_3_, 600 MHz): δ 1.51 (3H, s), 2.05 (3H, m), 3.88 (3H, s), 5.74 (1H, d, *J* = 11.2 Hz), 6.85 (1H, dd, *J* = 7.6 and 10.0 Hz), 7.55 (1H, m), 7.60 (2H, m), 7.65 (1H, m), 8.01 (1H, m), 8.05 (1H, d, *J* = 8.8 Hz), 8.10 (1H, d, *J* = 6.5 Hz) (Additional file 3). ^13^C NMR (CDCl_3_, 150 MHz): δ 22.5 q, 22.6 q, 65.0 q, 68.4 s**,** 105.4 **s,** 109.1 s**,** 115.5 d, 118.0 d, 126.7 d, 127.8 d, 128.2 d, 128.3 d, 130.2 d, 130.5 d, 130.6 d, 131.1 s, 151.4 s, 154.7 s, 157.3 s, 157.4 s, 174.7 s (Additional file 4). Oleic acid (**3**): white crystal. UV (EtOH): λ_max_ nm = 205. EI-MS (70 eV), *m*/*z* (% rel. int.): 282 [M]^+^ (3), 264 (14), 220 (3), 180 (4), 111 (21), 97 (44), 69 (76), 55 (100). ^1^H NMR (CDCl_3_, 600 MHz): δ 0.88 (3H, s), 1.29 (16H, m), 1.30 (2H, s), 1.33 (2H, s), 1.64 (2H, m), 2.03 (2H, m), 2.05 (2H, m), 2.35 (2H, s), 5.29 (1H, m), 5.34 (1H, m), 11.3 (1H, br) (Additional file 5). ^13^C NMR (CDCl_3_, 150 MHz): δ 14.1 q , 22.7 t, 24.8 t, 27.3 t, 29.0 t, 29.3 t, 29.4 t, 29.5 t, 29.6 t, 31.9 t, 34.2 t, 130.0 d, 130.2 d, 180.6 s (Additional file 6). Palmitic acid (**4**): white crystal. UV (EtOH): λ_max_ nm = 210. EI-MS (70 eV), *m*/*z* (% rel. int.): 256 [M]^+^ (100), 241 (4), 213 (20), 185 (10), 129 (32), 97 (20), 73 (58), 57 (46). ^1^H NMR (CDCl_3_, 600 MHz): δ 0.88 (3H, s), 1.29 (16H, m), 1.30 (2H, s), 1.33(2H, s), 1.64 (2H, m), 2.03 (2H, m), 2.05 (2H, m), 2.35 (2H, s), 11.6 (1H, br) (Additional file 7). ^13^C NMR (CDCl_3_, 150 MHz): δ 14.1 q, 22.7 t, 24.8 t, 27.3 t, 29.0 t, 29.3 t, 29.4 t, 29.5 t, 29.6 t, 31.9 t, 34.2 t, 179.8 s (Additional file 8). Pongamol (**5**): needle. UV (EtOH): λ_max_ nm = 260, 350. EI-MS (70 eV), *m*/*z* (% rel. int.): 294 [M]^+^ (52), 276 (19), 263 (24), 207 (22), 179 (62), 160 (100), 148 (97), 105 (19), 75 (91). ^1^H NMR (CDCl_3_, 600 MHz): δ 4.25 (3H, s), 7.01 (1H, *J* = 8.2 Hz), 7.15 (1H, s), 7.26 (1H, d, *J* = 2.2 Hz), 7.36 (2H, d, *J* = 0.5 Hz), 7.49 (1H, d, *J* = 1.6 Hz), 7.68 (2H, d, *J* = 6.5 Hz), 7.85 (1H, s), 7.92 (1H, m), 17.20 (1H, s) (Additional file 9). ^13^C NMR (CDCl_3_, 150 MHz): δ 61.8 q, 98.3 d, 103.6 d, 107.8 d, 108.1 s, 120.5 s, 122.5 d, 124.3 d, 126.1 d, 127.6 d, 127.6 d, 128.3 d, 130.2 s, 146.8 d, 153.0 s, 160.1 s, 185.4 s, 186.5 s (Additional file 10). Pongarotene (**6**): colorless solid. UV (EtOH): λ_max_ nm = 260, 330. EI-MS (70 eV), *m*/*z* (% rel. int.): 290 [M]^+^ (10), 205 (33), 187 (100), 176 (4), 160 (64), 145 (9), 131 (22), 121 (11), 69 (20). ^1^H NMR (CDCl_3_, 600 MHz): δ 6.16 (2H, s), 7.06 (1H, d, *J* = 8.3 Hz), 7.47 (1H, m), 7.67 (1H, d, *J* = 3.6 Hz), 7.74 (2H, d, *J* = 1.7 Hz), 7.84 (1H, dd, *J* = 1.7, 1.8 Hz), 8.07 (1H, dd, *J* = 2.2, 14.5 Hz), 8.20 (1H, m) (Additional file 11). ^13^C NMR (CDCl_3_, 150 MHz): δ 60.4 t, 103.3 d, 109.6 d, 111.6 s, 118.5 s, 119.5 d, 121.1 s, 122.7 d, 124.9 s, 126.1 d, 127.7 d, 130.5 d, 142.4 d, 148.0 s, 149.4 s, 155.5 s, 159.0 s, 175.2 s (Additional file 12).

### Larvicidal activity of test compounds

The toxicity of four flavonoids, eight fatty acids, four fatty acid esters, and two larvicides (temephos and fenthion) to third instar larvae from insecticide-susceptible *C. pipiens pallens* was evaluated by a direct-contact mortality bioassay (Table 3). Responses varied according to compound tested. Based on 24 h LC_50_ values, karanjin (14.61 mg/L) was the most toxic compound, followed by oleic acid (18.07 mg/L) and karanjachromene (18.74 mg/L). LC_50_ of linoleic acid, linolenic acid, pongamol, pongarotene, elaidic acid, and palmitic acid is between 20.15 and 34.50 mg/L. Low toxicity was produced by arachidic acid and behenic acid. Stearic acid and four fatty acid esters were ineffective. Overall, all of the constituents were less toxic than either temephos or fenthion. Mortality in the methanol–surfactant–water-treated controls for *C. pipiens pallens* larvae in this study was less than 2%.

**Table 3:** **Contact toxicity of four flavonoids, eight fatty acids, four fatty acid esters, and two larvicides to third instar** ***Culex pipiens pallens*** **larvae during a 24 h exposure**

**Compound**	**LC**_**50**_ **, mg/L (95% CL)**	**Slope ± SE**	**χ** ^**2a**^	***p*** **-value**
Karanjin (1)^b^	14.61 (12.11–17.81)	2.2 ± 0.30	1.41	0.999
Oleic acid (3)^b^	18.07 (15.55–20.52)	3.2 ± 0.41	1.80	0.997
Karanjachromene (2)^b^	18.74 (16.25–21.18)	3.3 ± 0.42	1.74	0.997
Linoleic acid	20.15 (17.13–24.44)	2.2 ± 0.29	1.17	0.999
Linolenic acid	21.34 (18.21–25.87)	2.3 ± 0.30	1.42	0.999
Pongamol (5)^b^	23.95 (20.68–27.34)	2.6 ± 0.33	1.26	0.999
Pongarotene (6)^b^	25.52 (22.14–29.16)	2.6 ± 0.34	1.54	0.999
Elaidic acid	28.22 (25.55–31.09)	3.9 ± 0.42	7.38	0.881
Palmitic acid (4)^b^	34.50 (36.36–40.72)	3.6 ± 0.48	2.55	0.999
Arachidic acid	54.91 (50.05–60.31)	3.9 ± 0.45	13.1	0.439
Behenic acid	68.76 (64.54–73.28)	6.5 ± 0.84	7.42	0.685
Stearic acid	>200			
Methyl oleate	>200			
Ethyl oleate	>200			
Methyl linoleate	>200			
Ethyl linoleate	>200			
Temephos	0.013 (0.010–0.016)	2.0 ± 0.30	2.40	0.935
Fenthion	0.027 (0.023–0.031)	3.5 ± 0.44	2.81	0.902

^a^Pearson’s chi-square goodness-of-fit test.

^b^Natural compounds isolated from *Millettia pinnata* seed in this study. The other 10 compounds were commercially organic pure compounds.

Toxic effects of all compounds on third instar larvae from insecticide-susceptible *A. aegypti* were likewise compared (Table 4). As judged by 24 h LC_50_ values, karanjin (16.13 mg/L) was most toxic and was less toxic than either temephos or fenthion. LC_50_ of oleic acid, karanjachromene, linoleic acid, linolenic acid, and pongamol was between 18.45 and 25.76 mg/L. LC_50_ of elaidic acid, pongarotene, and palmitic acid was between 32.16 and 42.96 mg/L. Low toxicity was obtained from arachidic acid and behenic acid. Stearic acid and four fatty acid esters were ineffective.

**Table 4:** **Contact toxicity of four flavonoids, eight fatty acids, four fatty acid esters, and two larvicides to third instar** ***Aedes aegypti*** **larvae during a 24 h exposure**

**Compound**	**LC**_**50**_ **, mg/L (95% CL)**	**Slope ± SE**	**χ** ^**2a**^	***p*** **-value**
Karanjin (1)^b^	16.13 (13.61–18.99)	2.2 ± 0.26	1.56	0.999
Oleic acid (3)^b^	18.45 (15.75–21.08)	3.0 ± 0.40	1.71	0.998
Karanjachromene (2)^b^	20.57 (17.69–23.57)	2.9 ± 0.40	2.02	0.996
Linoleic acid	21.28 (18.05–26.02)	2.1 ± 0.29	1.24	0.999
Linolenic acid	22.57 (19.31–27.41)	2.4 ± 0.31	1.01	0.999
Pongamol (5)^b^	25.76 (22.44–29.35)	2.7 ± 0.34	1.48	0.999
Elaidic acid	32.16 (29.15–35.62)	3.8 ± 0.44	3.13	0.997
Pongarotene (6)^b^	37.61 (33.96–41.50)	3.6 ± 0.44	4.88	0.977
Palmitic acid (4)^b^	42.96 (39.25–46.37)	4.6 ± 0.62	1.65	0.999
Arachidic acid	60.51 (55.09–67.01)	3.8 ± 0.45	15.4	0.283
Behenic acid	86.83 (81.18–92.83)	6.1 ± 1.20	4.07	0.944
Stearic acid	>200			
Methyl oleate	>200			
Ethyl oleate	>200			
Methyl linoleate	>200			
Ethyl linoleate	>200			
Temephos	0.015 (0.013–0.018)	2.6 ± 0.32	2.41	0.992
Fenthion	0.022 (0.019–0.024)	3.6 ± 0.43	2.61	0.989

^a^Pearson’s chi-square goodness-of-fit test.

^b^Natural compounds isolated from *Millettia pinnata* seed in this study. The other 10 compounds were commercially organic pure compounds.

Against third instar larvae from wild *A. albopictus* (Table 5), oleic acid (24 h LC_50_, 18.79 mg/L) was the most toxic compound, followed by karanjin (35.26 mg/L). These compounds were less toxic than either temephos or fenthion. LC_50_ of karanjachromene, pongamol, pongarotene, elaidic acid, linoleic acid, and linolenic acid was between 52.97 and 71.34 mg/L. Low toxicity was observed with palmitic acid, arachidic acid, and behenic acid. Stearic acid and four fatty acid esters were ineffective.

**Table 5:** **Contact toxicity of four flavonoids, eight fatty acids, four fatty acid esters, and two larvicides to third instar larvae from** ***Aedes albopictus*** **during a 24 h exposure**

**Compound**	**LC**_**50**_ **, mg/L (95% CL)**	**Slope ± SE**	**χ** ^**2a**^	***p*** **-value**
Karanjin (1)^b^	35.26 (31.01–39.54)	3.1 ± 0.41	3.36	0.996
Oleic acid (3)^b^	18.79 (16.23–21.33)	3.2 ± 0.41	2.25	0.994
Karanjachromene (2)^b^	52.97 (46.52–60.17)	2.7 ± 0.41	5.45	0.963
Pongamol (5)^b^	56.14 (49.50–64.69)	2.8 ± 0.33	8.42	0.815
Pongarotene (6)^b^	64.97 (57.47–72.53)	3.1 ± 0.43	3.64	0.994
Elaidic acid	66.35 (58.70–74.21)	3.1 ± 0.43	3.86	0.992
Linoleic acid	68.92 (57.12–80.61)	3.0 ± 0.78	1.02	0.996
Linolenic acid	71.34 (59.95–84.19)	2.9 ± 0.78	1.06	0.995
Palmitic acid (4)^b^	85.61 (78.31–93.69)	4.5 ± 0.63	2.47	0.991
Arachidic acid	95.01 (86.64–105.01)	3.5 ± 0.38	6.12	0.986
Behenic acid	105.41 (98.00–113.97)	4.9 ± 0.59	9.56	0.728
Stearic acid	>200			
Methyl oleate	>200			
Ethyl oleate	>200			
Methyl linoleate	>200			
Ethyl linoleate	>200			
Temephos	0.010 (0.009–0.011)	4.1 ± 0.46	1.66	0.998
Fenthion	0.033 (0.025–0.046)	1.5 ± 0.29	1.82	0.997

^a^Pearson’s chi-square goodness-of-fit test.

^b^Natural compounds isolated from *Millettia pinnata* seed in this study. The other 10 compounds were commercially organic pure compounds.

### Acetylcholinesterase inhibition

**Figure 3:**
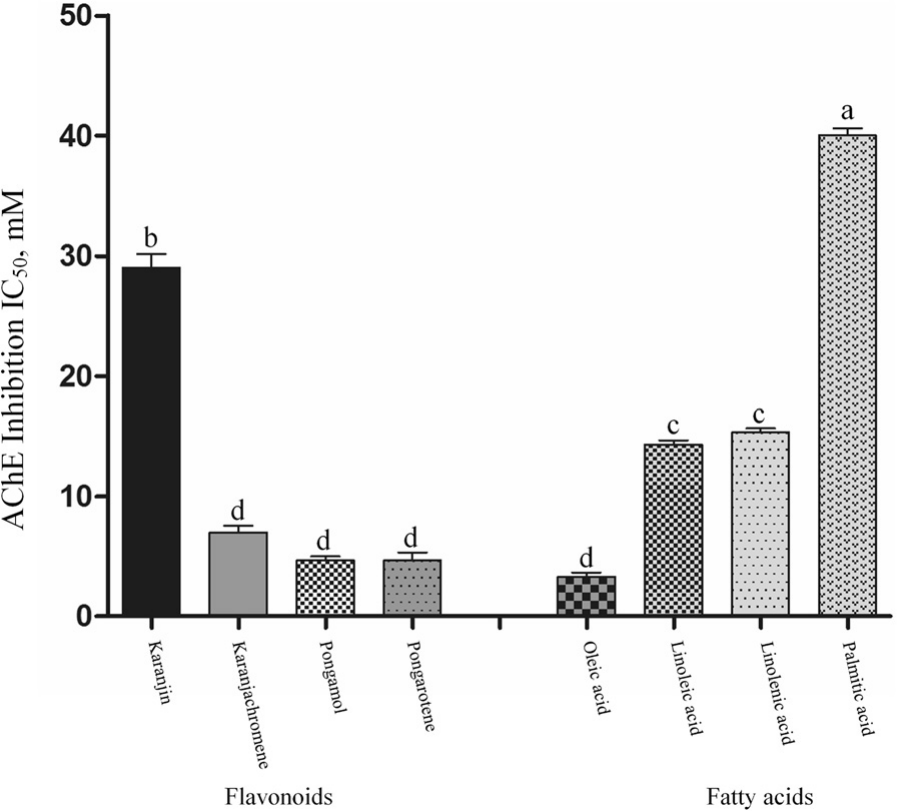
Inhibitory effect on acetylcholinesterase. Inhibition of acetylcholinesterase (AChE) extracted from third instar *Aedes aegyptii* larvae by four flavonoids, three unsaturated fatty acids, and one saturated fatty acid was measured by acetylthiocholine iodide hydrolysis at 30°C and pH 8.0. Each bar represents the mean ± standard error of triplicate samples of three independent experiments. (^*^ *p* = 0.05, according to Bonferroni multiple-comparison method).

Because of no inhibitory effects of arachidic acid, behenic acid, and elaidic acid on AChE (IC_50_, >50 mM) in preliminary test results, the *in vitro* AChE inhibitory activity of four flavonoids, three unsaturated fatty acids, and one saturated fatty acid was investigated using AChE from *A. aegypti* larvae (Figure 3). Based on IC_50_ values, there were significant differences (*F* = 155.49; df = 8, 18; p < 0.0001) in the inhibition of AChE by the test compounds. Karanjachromene, pongarotene, pongamol, and oleic acid were the most potent AChE inhibitors (IC_50_, 3.3–5.9 mM). Linoleic acid (IC_50_, 19.4 mM) and linolenic acid (21.4 mM) were significantly more pronounced at inhibiting AChE than karanjin (28.4 mM). The AChE inhibitory activity of palmitic acid was the lowest of any of the compounds examined.

### Effect on cyclic AMP production

**Figure 4:**
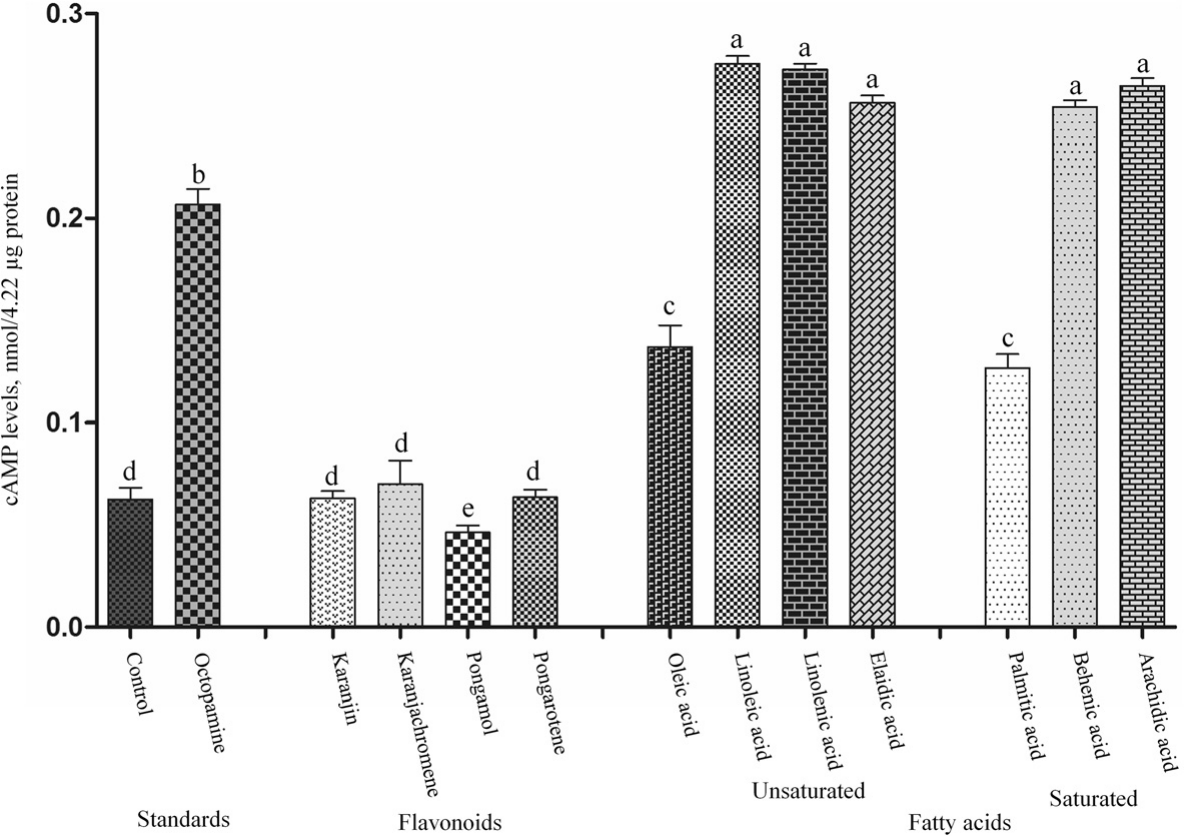
Effect on cyclic AMP levels. A whole body homogenate from third instar *Aedes aegypti* larvae was assayed for adenylate cyclase activity, as described in ‘Materials and Methods’ section, in the presence of 100 μM of four flavonoids, four unsaturated fatty acids, and three saturated fatty acids. The effects of the test compounds on cAMP levels of the homogenate were compared with those induced by octopamine (100 μM) alone. Data were expressed as nmol/4.22 μg protein. Each bar represents the mean ± standard error of duplicate samples of three independent experiments. (^*^ *p* = 0.05, according to Bonferroni multiple-comparison method).

The effects of four flavonoids, four unsaturated fatty acids, and three saturated fatty acids on cAMP levels of whole body homogenates from third instar *A. aegypti* larvae were examined and compared with those induced by octopamine alone (Figure 4). There were significant differences (*F* = 1641.51; df = 12, 26; p < 0.0001) in the cAMP levels by the test compounds. At a concentration of 100 μM, the cAMP levels induced by linoleic acid, linolenic acid, elaidic acid, behenic acid, and arachic acid were significantly higher than that induced by octopamine. The cAMP levels induced by the other four flavonoids and two fatty acids oleic acid and palmitic acid were significantly lower than that induced by octopamine.

### Efficacy of experimental liquid formulations

The control efficacy of 12 liquid formulations significantly differed toward *C. pipiens pallens* (*F* = 556.94; df = 6, 14; p < 0.0001), *A. aegypti* (*F* = 795.42; df = 8, 18; p < 0.0001), and *A. albopictus* (*F* = 1564.84; df = 8, 18; p < 0.0001) (Table 6). *M. pinnata* seed methanol extract applied as 10% liquid (MPS-ME-10) and seed hydrodistillate applied as 10% liquid (MPS-HD-10) provided 100% mortality toward three mosquito species larvae. The lethalities of the MPS-ME-7.5 and MPS-HD-7.5 were 100, 100 and 82% and 100, 100 and 92% toward *C. pipiens pallens*, *A. aegypti*, and *A. albopictus*, respectively. A commercial temephos 200 g/L treatment resulted in 100% mortality toward three mosquito species larvae.

**Table 6:** **Effectiveness of 12 experimental liquid formulations containing** ***Millettia pinnata*** **seed methanol extract, seed hydrodistillate, and commercial temephos emulsifiable concentrate toward larvae of three mosquito species during a 24 h exposure**

	**Mortality, % (± SE)**		
**Formulation (%)**	***C. pipiens pallens***	***A. aegypti***	***A. albopictus***
MPS-ME-0.5	35 ± 2.9 e*	25 ± 2.9 f*	0 g*
MPS-ME-1	59 ± 2.4 d	40 ± 2.9 e	17 ± 1.7 f
MPS-ME-2.5	73 ± 1.7 c	67 ± 1.7 d	37 ± 3.3 e
MPS-ME-5	97 ± 1.7 b	85 ± 2.9 c	58 ± 1.7 d
MPS-ME-7.5	100 a	100 a	82 ± 1.7 c
MPS-ME-10	100 a	100 a	100 a
MPS-HD-0.5	38 ± 1.7 e	26 ± 1.7 f	0 g
MPS-HD-1	68 ± 1.7 d	45 ± 2.9 e	17 ± 1.7 f
MPS-HD-2.5	100 a	68 ± 2.9 d	32 ± 1.7 e
MPS-HD-5	100 a	96 ± 3.3 b	62 ± 2.4 d
MPS-HD-7.5	100 a	100 a	92 ± 1.7 b
MPS-HD-10	100 a	100 a	100 a
Temephos 200 g/L EC	100 a	100 a	100 a

MPS-ME, *Millettia pinnata* seed methanol extract; MPS-HD, *Millettia pinnata* seed hydrodistillate; EC, emulsifiable concentrate.

*Means followed by the same letter in the column are not significantly different (p = 0.05, Bonferroni method).

## Discussion

Certain plant preparations can be developed into products suitable for integrated vector management because they can be selective, biodegrade to nontoxic products, have few harmful effects on nontarget organisms, and are environmentally nonpersistent [16-18]. They also can be used in conjunction with biological control [18]. Sukumar et al. [16] has pointed out that the most promising botanical mosquito control agents are plants in the families Asteraceae, Cladophoraceae, Lamiaceae, Meliaceae, Oocystaceae, and Rutaceae. The efficacy of various botanical extracts and their fractions toward larvae of various mosquito species (LC_50_, 2.6–44400 mg/L) has been well documented by Shaalan et al. [17], although the activity can vary significantly depending on plant species, plant tissue, age of plant, solvent used in extraction, and mosquito species [16]. In the current study, *M. pinnata* seed methanol extract and seed hydrodistillate exhibited good larvicidal activity against *C. pipiens pallens*, *A. aegypti*, and *A. albopictus* (LC_50_, 24.19–61.30 mg/L and 27.38–47.99 mg/L), although this plant belongs to the family Fabaceae. *M. pinnata* contains abundant flavonoid metabolites such as chromenoflavones, furanoflavones, furanoflavonols, furanochalcones, and pyranochalcones [24,25].

Many plant preparations manifest toxicity to different mosquito species larvae [16,17,38] and have been proposed as potential alternatives to the conventional larvicides. Larvicidal constituents derived from plants include alkaloids (e.g., pellitorine, guineensine, pipercide, and retrofractamide A, LC_50_ 0.004–0.86 mg/L [39]; piperonaline, LC_50_ 0.21–0.25 mg/L [40]), coumarins (e.g., imperatorin and osthole, LC_50_ 2.88 and 3.14 mg/L [22]), phenylpropanoids (e.g., methyleugenol and α-asarone, LC_50_ 10.49 and 26.99 mg/L [21]; ethyl cinnamate and ethyl *p*-methoxycinnamate, LC_50_ 12.3 and 20.7 mg/L [28]), terpenoids (e.g., 47 terpenes, LC_50_ 9.33–147.91 mg/L [41]; quassin, LC_50_ 6.0 mg/L [42]; labda-8(17)-diene-15,16-dial, LC_50_ < 10 mg/L [43]), neolignans (e.g., conocarpan, eupomatenoid-5, and eupomatenoid-6, LC_50_ < 10 mg/L [44]), cyanogenic glycoside (e.g., dhurrin, LC_50_ 1.12 mg/L [45]), polyacetylene (e.g., capillin, LC_50_ < 10 mg/L [46]), lactones (e.g., goniothalmin, LC_50_ 0.87–25.95 mg/L [47]; butenolides 1 and 2, LC_50_ 0.41 and 0.47 mg/L [48]), acetylenic alcohols (e.g., falcarinol and falcarindiol, LC_50_ 3.49 and 6.51 mg/L [49]), and phenols (e.g., 4-butoxymethylphenol, LC_50_ 0.05 mg/L [50]).

In the current study, the larvicidal principles of *M. pinnata* seed were determined to be the furanoflavonoid karanjin (1), the pyranoflavonoid karanjachromene (2), the unsaturated fatty acids oleic acid (3), the saturated fatty acids palmitic acid (4), the dihydrochalcone flavonoid pongamol (5), and the rotenoid flavonoid pongarotene (6) from seed extract, as well as the unsaturated fatty acids oleic acid, elaidic acid, linoleic acid and linolenic acid and the saturated fatty acids palmitic acid, arachidic acid and behenic acid from seed hydrodistillate. The interpretations of proton and carbon signals of compounds 1, 2, 3, 4, 5, and 6 were largely consistent with those of Katekhaye et al. [51], Koysomboon et al. [52], Yang et al. [53], Ragona et al. [54], Parmar et al. [55], and Simin et al. [56], respectively. LC_50_ of the four flavonoids and the seven fatty acids was between 14.61 and 64.97 mg/L and between 18.07 and 105.41 mg/L toward three mosquito species larvae, respectively, although LC_50_ of the natural compounds stated previously is between 0.004 and 147.91 mg/L. Karanjin, karanjachromene, and oleic acid were highly effective toward *C. pipiens pallens* and *A. aegypti* larvae, whereas karanjin and karanjachromene were relatively less effective toward *A. albopictus* larvae. Oleic acid was highly effective toward *A. albopictus* larvae. In addition, the 10% liquids containing *M. pinnata* seed methanol extract and seed hydrodistillate resulted in complete control toward three mosquito species larvae and the larvicidal activity of the liquids was comparable to that of commercial temephos 200 g/L EC. Treatment with the 7.5% extract and seed hydrodistillate liquids resulted in complete control toward *C. pipiens pallens* and *A. aegypti* larvae and 82 and 92% mortality toward *A. albopictus* larvae, respectively. This susceptibility difference might be attributable to the development of insecticide resistance in wild *A. albopictus* collected near rice paddy fields and cowsheds with varying histories of insecticide use. This original finding indicates that materials derived from *M. pinnata* seed may hold promise for the development of novel and effective mosquito larvicides toward mosquito field populations. Karanjin is commercialized as an insecticide/acaricide (20 g/L EC) for the control of mites, scales, and chewing and sucking insect pests in a wide range of agricultural crops and ornamentals [57].

QSAR analysis of phytochemicals toward mosquito larvae has been well noted [22,39]. However, limited information is available on larvicidal activity of fatty acids. Unsaturated fatty acids (oleic acid, linoleic acid, and linolenic acid) were reported to be more toxic than unsaturated fatty acids (myristic acid, palmitic acid, and stearic acid) toward *A. albopictus* larvae [58]. In the current study, the unsaturated fatty acids (oleic acid, linoleic acid, linolenic acid, and elaidic acid) were more pronounced in toxicity to three mosquito species larvae than the saturated fatty acids (arachidic acid, behenic acid, palmitic acid, and stearic acid). The toxicity of oleic acid, linoleic acid, and linolenic acid did not differ significantly. Oleic acid was more toxic than elaidic acid, the *trans* isomer of oleic acid. Palmitic acid was more toxic than arachidic acid and behenic acid. Two FAMEs methyl oleate and methyl linoleate and two fatty acid ethyl esters ethyl oleate and ethyl linoleate were ineffective. These findings indicate that it might be possible to use unsaturated fatty acids as environmentally safe and effective larvicides. QSAR indicates that structural characteristics, such as the degree of saturation, the side chain length of fatty acid, and the geometric isomerism, appear to play a role in determining the fatty acid toxicity to mosquito larvae.

Investigations on the modes of action of naturally occurring compounds may provide useful information for the development of biorational insecticides with novel target sites and for future resistance management [17,18]. The modes of insecticidal action of naturally occurring compounds are mainly due to AChE inhibition and interference with the octopaminergic system [18]. Certain terpenoids such as pulegone-1,2-epoxide and 1,8-cineole inhibit AChE from *A. aegypti* larvae (IC_50_, 1.45–74.33 mM) [41], housefly and Madagascar roach [59], head louse (IC_50_, 77 mM) [60], and three stored-product insect pests [61]. Ryan and Byrne [62] reported a relationship between insecticidal and electric eel AChE inhibitory activities of terpenoids, whereas no direct correlation between insect toxicity and AChE inhibition by terpenoids was also reported [59,60]. The octopaminergic and gamma aminobutyric acid receptors have also been suggested as novel target sites for some monoterpenoid essential oil constituents in *Helicoverpa armigera* (Hübner 1809 [63]) [19] and *Drosophila melanogaster* Meigen 1830 [64], [20], respectively.

In the current study, no correlation was found between contact toxicity and AChE inhibition. The flavonoids karanjachromene, pongamol, and pongarotene strongly inhibited mosquito larval AChE. Karanjin was 4.8-fold less pronounced at inhibiting AChE than karanjachromene, although the toxicity of the two flavonoids did not differ significantly. The two flavonoids had no effect on cAMP levels. These findings indicate that AChE is the main site of action of karanjin and karanjachromene. The unsaturated fatty acid oleic acid and the saturated fatty acid palmitic acid strongly and weakly inhibited AChE, respectively. The cAMP levels induced by the fatty acids were lower than that induced by octopamine alone. These findings indicate that AChE is the main site of action of oleic acid and palmitic acid. The unsaturated fatty acid elaidic acid and the saturated fatty acids arachidic acid and behenic acid were ineffective at inhibiting AChE and caused a considerable increase in cAMP levels, indicating that the mechanism of insecticidal action of elaidic acid, arachidic acid, and behenic acid might be due to interference with the octopaminergic system. Linoleic acid and linolenic acid moderately inhibited AChE and caused a considerable increase in cAMP levels. This finding indicates that linoleic acid and linolenic acid might act on both AChE and octopaminergic receptor. Detailed tests are needed to fully understand the exact modes of action of the flavonoids and the fatty acids. It has been also reported that karanjin suppresses ecdysteroids and, thereby, it acts as an insect growth regulator and antifeedant [57].

## Conclusion

The *M. pinnata* seed-derived products containing the four natural flavonoids and two natural fatty acids described could be useful as larvicides in the control of mosquito populations. This plant is a fast-growing leguminous tree with the potential for high seed production in Indian subcontinent, south-east Asia, and humid tropical regions of the world [65]. There is, therefore, a potential source of the seed extract or hydrodistillate available as an eco-product. For practical use of the products as novel mosquito larvicides to proceed, further research is needed to establish their safety to humans, although historically, *M. pinnata* has been used in India and neighboring regions as a source of traditional medicines and animal fodder [25,65,66]. In addition, their effects on non-target aquatic organisms including larvivorous fishes and the aquatic environment need to be established. Lastly, detailed tests are needed to understand how to improve larvicidal potency and stability for eventual commercial development.

## Additional files

Additional file 1:
^**1**^ **H NMR (CDCl**_**3**_ **, 600 MHz) spectrum of karanjin (1).**
Additional file 2:
^**13**^ **C NMR (CDCl**_**3**_ **, 150 MHz) spectrum of karanjin (1).**
Additional file 3:
^**1**^ **H NMR (CDCl**_**3**_ **, 600 MHz) spectrum of karanjachromene (2).**
Additional file 4:
^**13**^ **C NMR (CDCl**_**3**_ **, 150 MHz) spectrum of karanjachromene (2).**
Additional file 5:
^**1**^ **H NMR (CDCl**_**3**_ **, 600 MHz) spectrum of oleic acid (3).**
Additional file 6:
^**13**^ **C NMR (CDCl**_**3**_ **, 150 MHz) spectrum of oleic acid (3).**
Additional file 7:
^**1**^ **H NMR (CDCl**_**3**_ **, 600 MHz) spectrum of palmitic acid (4).**
Additional file 8:
^**13**^ **C NMR (CDCl**_**3**_ **, 150 MHz) spectrum of palmitic acid (4).**
Additional file 9:
^**1**^ **H NMR (CDCl**_**3**_ **, 600 MHz) spectrum of pongamol (5).**
Additional file 10:
^**13**^ **C NMR (CDCl**_**3**_ **, 150 MHz) spectrum of pongamol (5).**
Additional file 11:
^**1**^ **H NMR (CDCl**_**3**_ **, 600 MHz) spectrum of pongarotene (6).**
Additional file 12:
^**13**^ **C NMR (CDCl**_**3**_ **, 150 MHz) spectrum of pongarotene (6).**
