# Supplementary figures and images for: Larvicidal activity and possible mode of action of four flavonoids and two fatty acids identified in Millettia pinnata seed toward three mosquito species
Source: Parasit Vectors. 2015 Apr 19;8:237. doi: 10.1186/s13071-015-0848-8 (PMC4410478; doi:10.1186/s13071-015-0848-8)

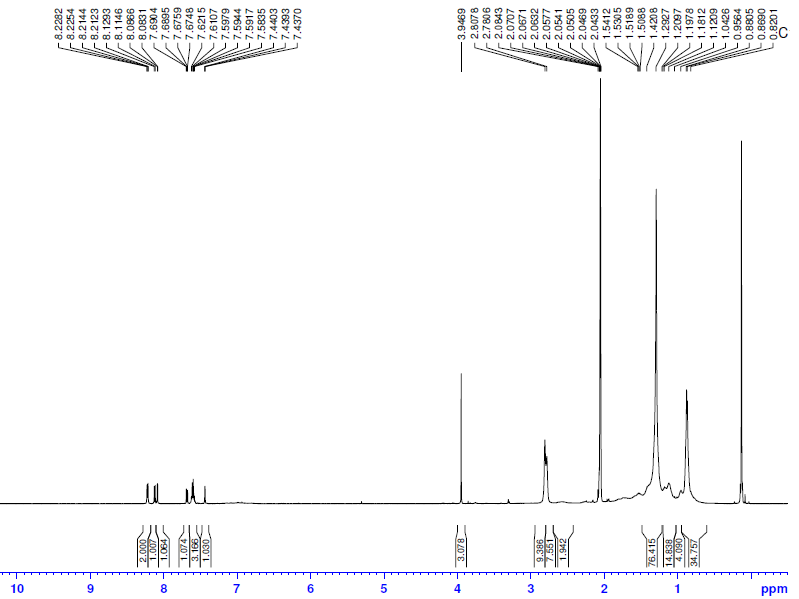

Supplement: Additional file 1: — 1 H NMR (CDCl 3 , 600 MHz) spectrum of karanjin (1). [file 13071_2015_848_MOESM1_ESM.tif]

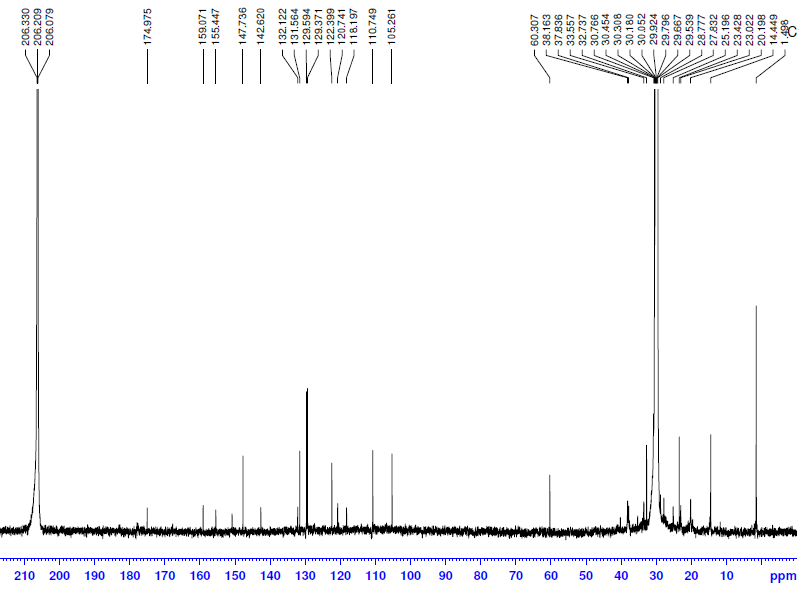

Supplement: Additional file 2: — 13 C NMR (CDCl 3 , 150 MHz) spectrum of karanjin (1). [file 13071_2015_848_MOESM2_ESM.tif]

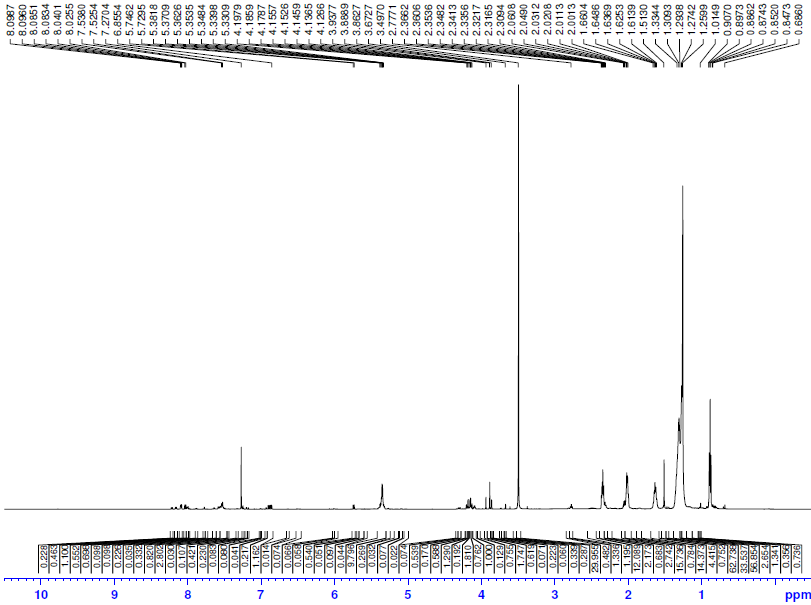

Supplement: Additional file 3: — 1 H NMR (CDCl 3 , 600 MHz) spectrum of karanjachromene (2). [file 13071_2015_848_MOESM3_ESM.tif]

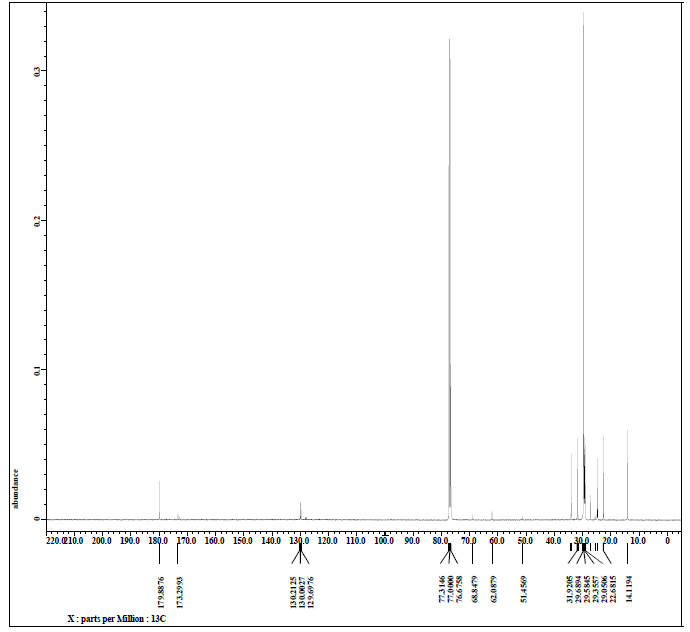

Supplement: Additional file 4: — 13 C NMR (CDCl 3 , 150 MHz) spectrum of karanjachromene (2). [file 13071_2015_848_MOESM4_ESM.tif]

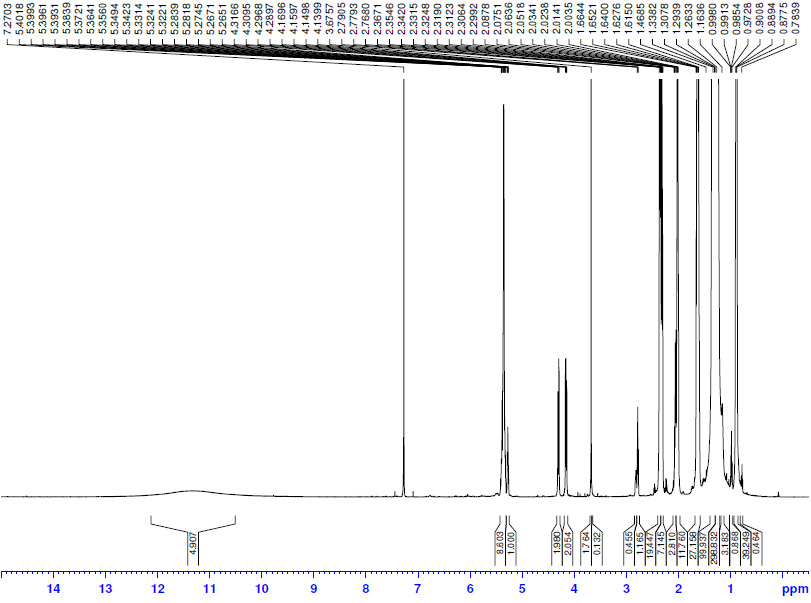

Supplement: Additional file 5: — 1 H NMR (CDCl 3 , 600 MHz) spectrum of oleic acid (3). [file 13071_2015_848_MOESM5_ESM.tif]

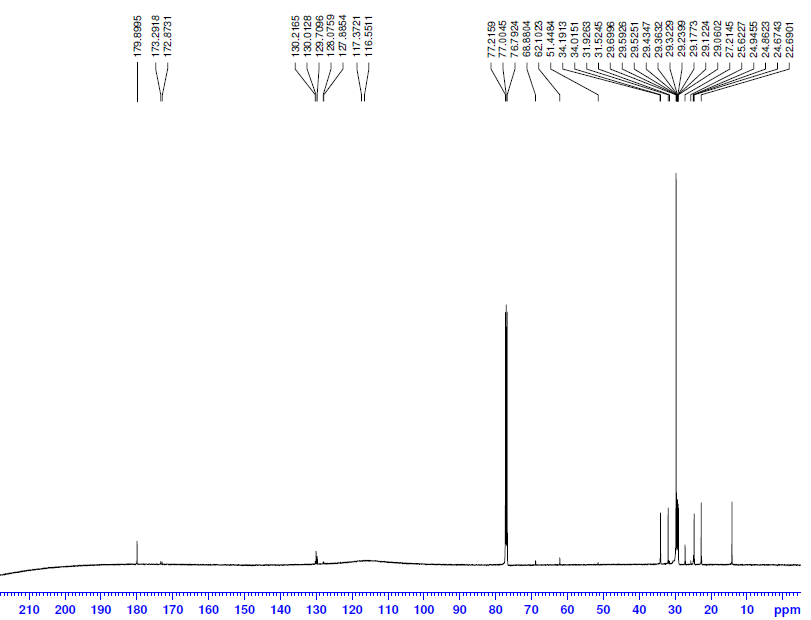

Supplement: Additional file 6: — 13 C NMR (CDCl 3 , 150 MHz) spectrum of oleic acid (3). [file 13071_2015_848_MOESM6_ESM.tif]

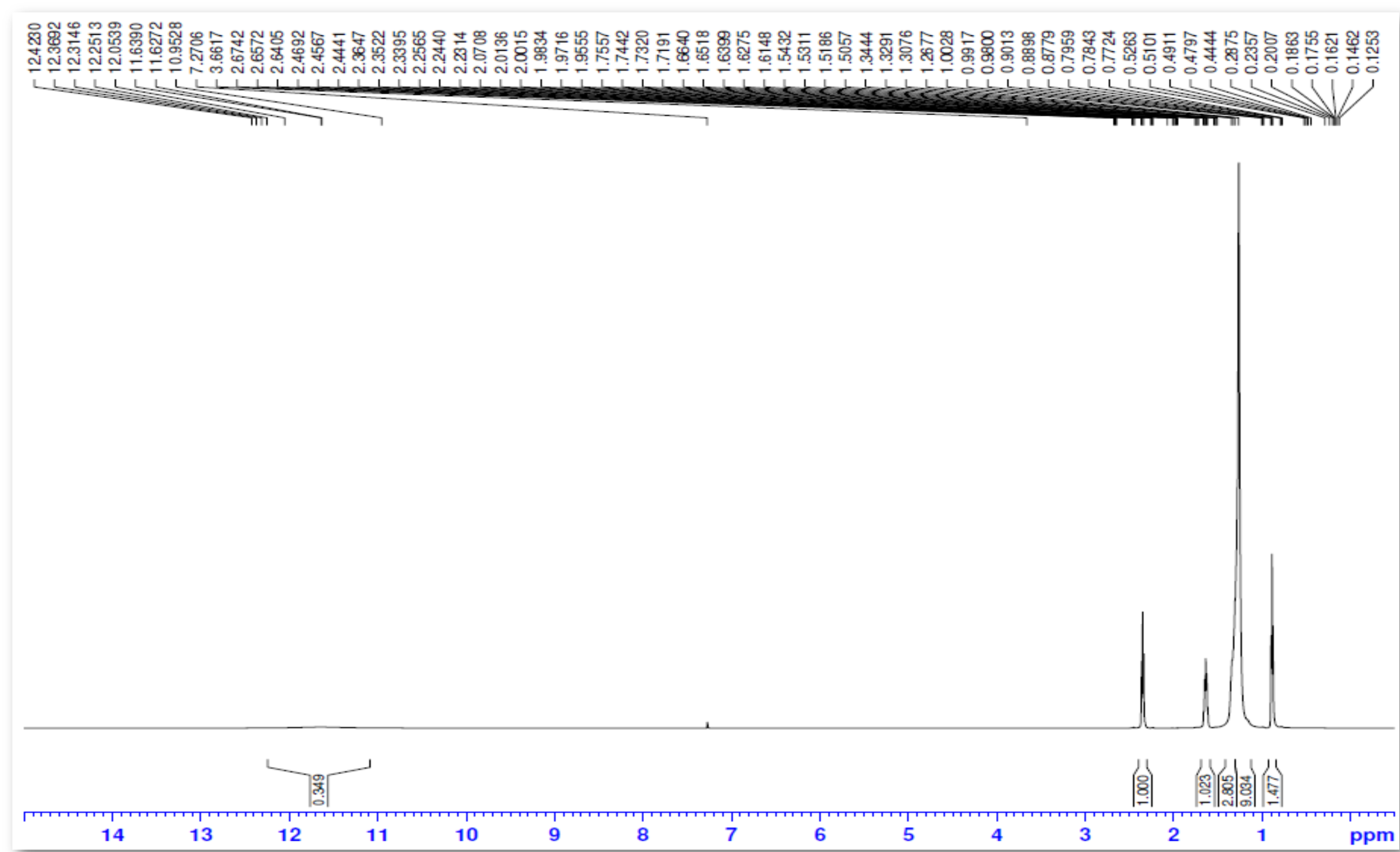

Supplement: Additional file 7: — 1 H NMR (CDCl 3 , 600 MHz) spectrum of palmitic acid (4). [file 13071_2015_848_MOESM7_ESM.tif]

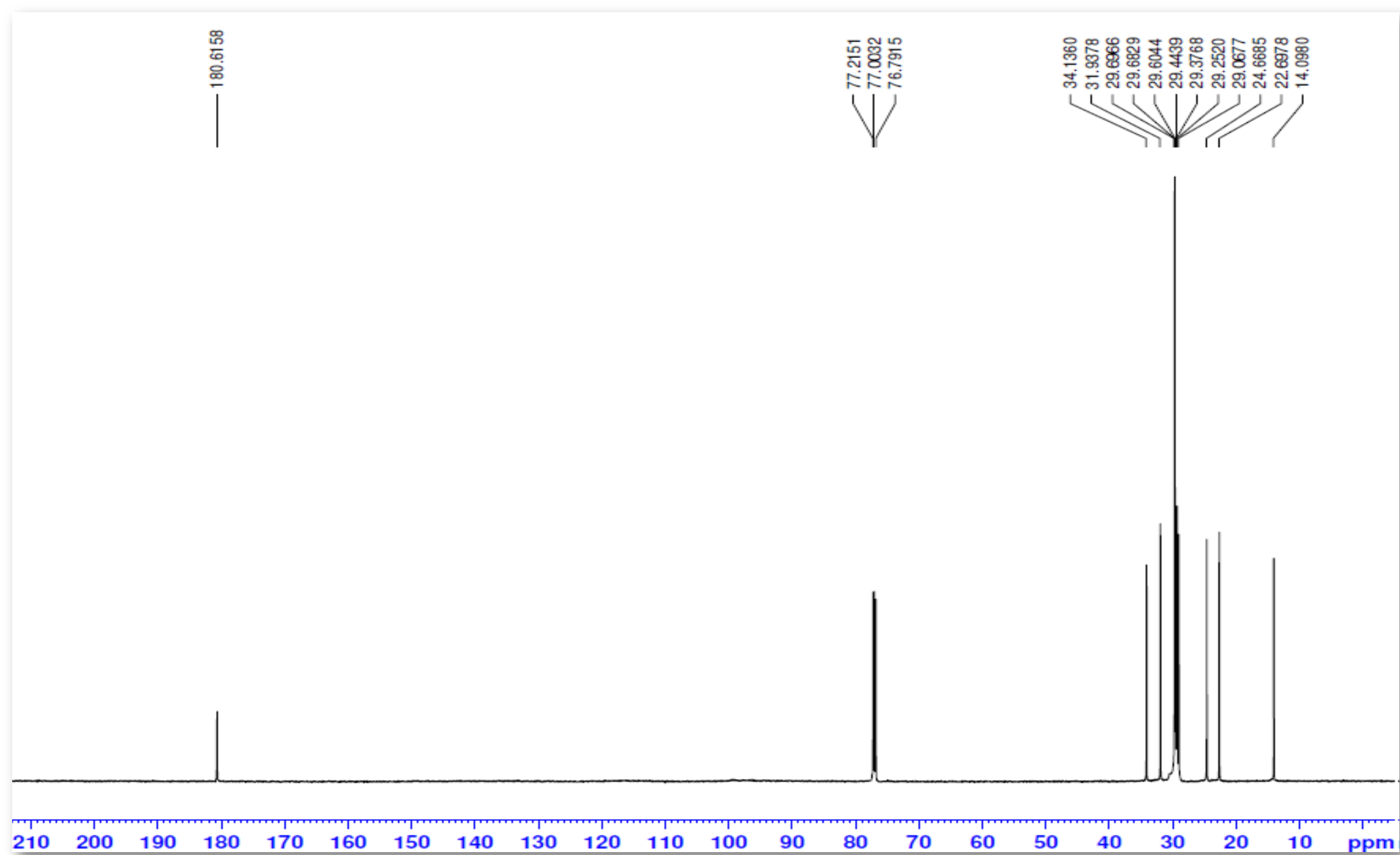

Supplement: Additional file 8: — 13 C NMR (CDCl 3 , 150 MHz) spectrum of palmitic acid (4). [file 13071_2015_848_MOESM8_ESM.tif]

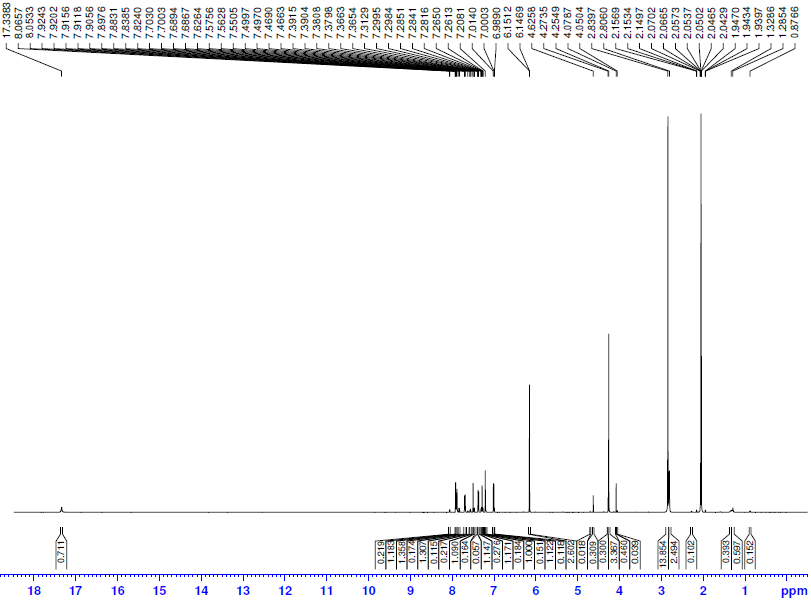

Supplement: Additional file 9: — 1 H NMR (CDCl 3 , 600 MHz) spectrum of pongamol (5). [file 13071_2015_848_MOESM9_ESM.tif]

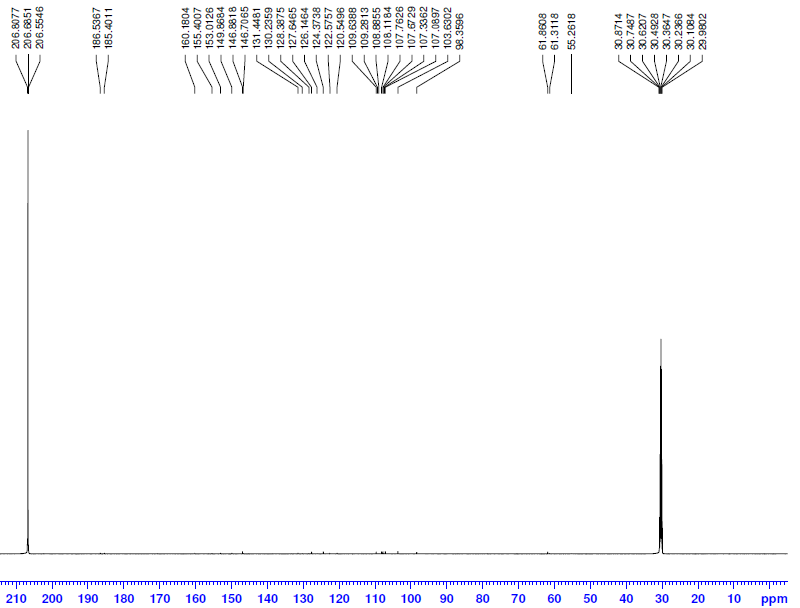

Supplement: Additional file 10: — 13 C NMR (CDCl 3 , 150 MHz) spectrum of pongamol (5). [file 13071_2015_848_MOESM10_ESM.tif]

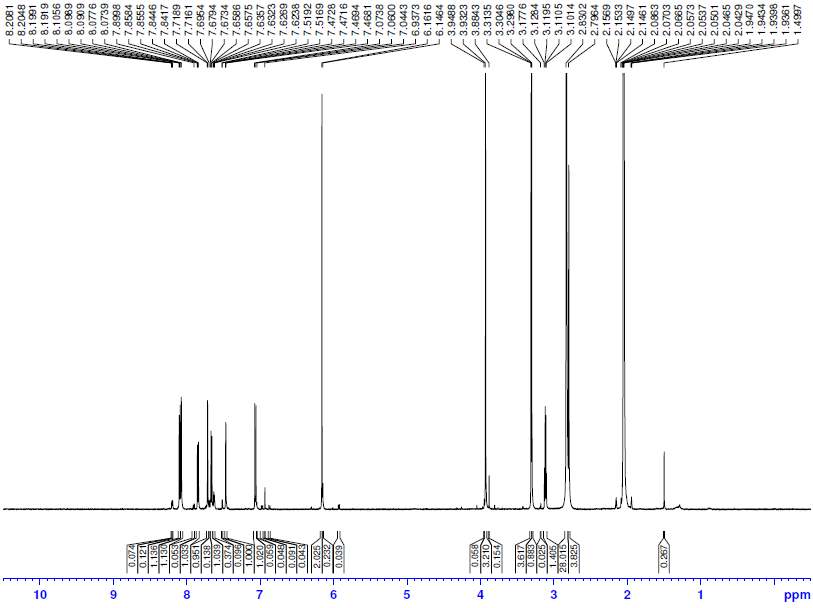

Supplement: Additional file 11: — 1 H NMR (CDCl 3 , 600 MHz) spectrum of pongarotene (6). [file 13071_2015_848_MOESM11_ESM.tif]

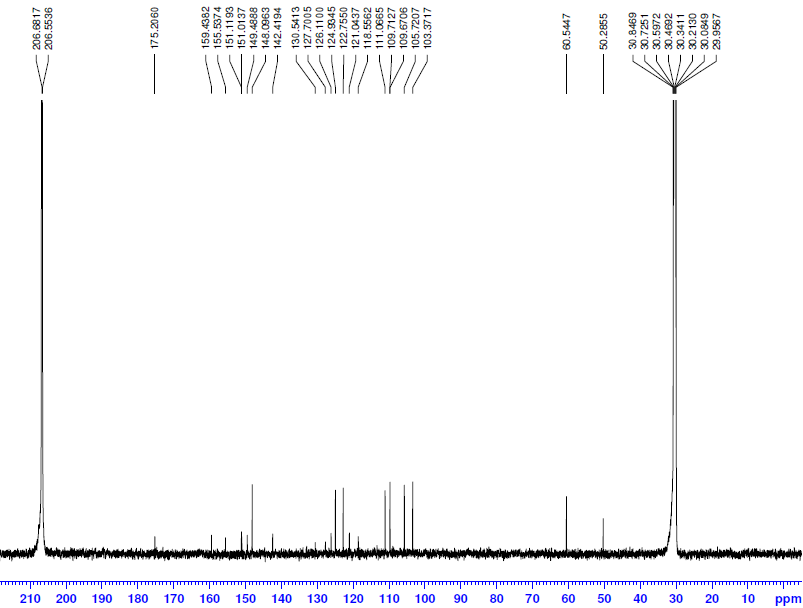

Supplement: Additional file 12: — 13 C NMR (CDCl 3 , 150 MHz) spectrum of pongarotene (6). [file 13071_2015_848_MOESM12_ESM.tif]
